# Galectin-3—A Multifunctional Molecule and a Key Player in Health and Disease

**DOI:** 10.3390/molecules31101694

**Published:** 2026-05-17

**Authors:** Alina Lupu (Surlea), Ticuta Negreanu-Pirjol, Laura Olariu, Bogdan-Stefan Negreanu-Pirjol, Sanda Jurja, Anca Cristina Lepadatu, Mihaela Basa, Natalia Rosoiu

**Affiliations:** 1 Institute of Doctoral Studies, Doctoral School of Applied Sciences, Domain Biology, “Ovidius” University of Constanta, 58, Ion Voda Street, 900573 Constanta, Romanianatalia_rosoiu@yahoo.com (N.R.); 2 Faculty of Pharmacy, “Ovidius” University of Constanta, 6, Capitan Aviator Al. Serbanescu Street, Campus, Building C, 900470 Constanta, Romania; 3 Academy of Romanian Scientists, Biological Sciences Section, 3, Ilfov Street, 050044 Bucharest, Romania; olariulaura@yahoo.com; 4 S.C. Biotehnos S.A., Gorunului Street, No. 3-5, 075100 Bucharest, Romania; 5 Faculty of Medicine, “Ovidius” University of Constanta, 1, University Alley, Campus, Building B, 900470 Constanta, Romania; sanda.jurja@365univ-ovidius.ro; 6 Faculty of Natural Sciences and Agricultural Sciences, “Ovidius” University of Constanta, 1, University Alley, Campus, Building B, 900470 Constanta, Romania; anca.lepadatu@365.univ-ovidius.ro; 7 Medical Analysis Laboratory, “Alexandru Gafencu” Military Emergency Hospital of Constanta, 96, Mamaia Bvd., 900527 Constanta, Romania; mihaela_basa@yahoo.com

**Keywords:** galectins, Galectin-3, proinflammatory, anti-inflammatory, biomarkers, cardiovascular diseases, chronic diseases, cancer

## Abstract

Galectins are a family of proteins that belong to one of the most widespread classes of lectins found in all organisms. They are found intracellularly in various structures or secreted into the extracellular space, where they are involved in mediating cellular biological processes such as growth, function, interaction and response. They are known for their ability to bind to carbohydrates, specifically those containing beta-galactose. This narrative review outlines the human galectins, emphasizing Galectin-3, a β-galactoside-binding protein with numerous pleiotropic regulatory activities; it mediates various biological processes. Galectin-3 is an extensively studied member due to its broad implications in health and disease and we highlight the need for deeper insights into the molecular mechanisms and clinical implications of Galectin-3 as well as the context-dependent mechanisms through which Galectin-3 influences diverse physiological and pathological processes. Literature syntheses and scientific research, as well as clinical and experimental studies, were investigated regarding the biological and pathophysiological actions and implications of Galectin-3 in diseases. The study presents the structure, characteristics, roles, activities, and proinflammatory actions (as a mediator) and anti-inflammatory actions (as a modulator) of Galectin-3, as well as the main groups of diseases in which it is involved or associated with cardiovascular diseases, cancer, organ fibrosis, and metabolic diseases. The results emphasized the potential of Galectin-3 as an important molecule and highlighted the necessity for further research to improve its approach. This narrative review provides new insights into identifying its functions and the future development and rational design of Galectin-3-directed research strategies.

## 1. Introduction

Galectins comprise a multifunctional family of carbohydrate-binding proteins involved in a wide spectrum of physiological and pathological processes. Initially described as β-galactoside-binding proteins, they are now recognized as important regulators of diverse biological mechanisms, including inflammation, fibrosis, immune responses, and tissue remodeling [1].

Galectins are structurally classified into three groups [2]:

1. Chimera-type group, containing only Galectin-3;
2. Prototype group, including Galectin-1, -2, -5, -7, -10, -11, -13, -14, -15, and -16;
3. Tandem-repeat group, including Galectin-4, -6, -8, -9, and -12.

Interest in this protein family has grown considerably, particularly in Galectin-3, owing to its broad involvement in the pathophysiology of human disease and its diverse roles in physiological regulation, cellular differentiation, and transformation [3].

Over the past few decades, Galectin-3 has evolved from a lectin primarily studied for its structural and binding properties into a clinically relevant molecule with recognized roles in inflammation, fibrosis, immune regulation, and tumor biology. More recent studies have further strengthened its relevance by highlighting its dual value as both a mechanistic mediator of disease and a promising biomarker or therapeutic target across multiple pathological settings [4,5,6]. Accordingly, it has attracted increasing interest as a potential therapeutic target, including in cancer-related settings [7].

Beyond its clinical relevance as a potential biomarker, Galectin-3 is increasingly regarded as an active mediator of disease pathogenesis. Current evidence implicates Galectin-3 in molecular pathways including NF-κB-associated inflammatory signaling, transforming growth factor-β (TGF-β)-mediated fibrotic responses, and extracellular matrix remodeling [5]. Nevertheless, several important gaps remain unresolved, such as the limited availability of longitudinal data, the heterogeneity of analytical methods used to quantify Galectin-3, and the lack of standardized disease-specific thresholds for its clinical interpretation. These limitations underscore the need for a narrative review of the available evidence.

The main aim of this review is to describe the roles of Galectin-3 in clinical conditions associated with inflammation and fibrosis and to examine its pathophysiological implications in several human disorders.

This review underlines the involvement of Galectin-3 in cardiovascular diseases, metabolic disorders, renal diseases, digestive diseases, neuroinflammation and neurodegenerative diseases, and endometriosis, while highlighting current research directions. The paper is focused on the molecular mechanisms and biological roles in several diseases. Finally, it emphasizes the possible role of Galectin-3 for roles as a biomarker or for further target therapy research.

## 2. Methodology and Search Strategy

### 2.1. Review Design and Reporting Framework

This study was designed as a narrative review with a systematic structure, examining the diagnostic, prognostic, pathophysiological, and therapeutic relevance of Galectin-3 in human diseases. The review was conducted and reported following the key organizational principles of the Preferred Reporting Items for Systematic Reviews and Meta-Analyses (PRISMA) 2020 framework [8], adapted to the scope of a narrative synthesis.

### 2.2. Review Question and PICO Framework

The main review question was formulated as follows: “What is the clinical and pathophysiological relevance of Galectin-3 in human diseases?”

Because this review addresses a biomarker and disease-associated mediator rather than a therapeutic intervention alone, an adapted PICO framework was used to define the review question.

- Population (P): human subjects or human-derived clinical samples from patients with the predefined disease categories: cardiovascular, metabolic, renal, digestive, gynecological, neurological, neurodegenerative, and ophthalmological disorders;
- Index factor/Exposure (I): assessment of Galectin-3 expression, circulating levels, tissue localization, or disease-related biological activity;
- Comparator (C): healthy controls, disease-free controls, patients with different disease severity, or studies without an explicit comparator but with clinically relevant Galectin-3-associated findings;
- Outcomes (O): diagnostic value, prognostic significance, association with disease severity or progression, mechanistic involvement in inflammation or fibrosis, and potential therapeutic relevance.

### 2.3. Eligibility Criteria

Studies were considered eligible if they met all of the following inclusion criteria: they were published in English, available in full text, published within the predefined time interval of 2020–2025, and focused on Galectin-3 in relation to human diseases. In addition, eligible studies were required to report data relevant to at least one predefined outcome, including the diagnostic, prognostic, mechanistic, inflammatory, fibrotic, or therapeutic implications of Galectin-3. Original studies, systematic reviews, meta-analyses, and narrative reviews relevant to the review question were all considered for inclusion.

Studies were excluded if they were duplicate records, were not primarily focused on Galectin-3, or addressed other members of the galectin family without providing relevant data on Galectin-3. Non-human studies lacking direct translational relevance to human disease were also excluded, as were publications without accessible full text, non-English publications, and conference abstracts, editorials, letters, commentaries, book chapters, and proceedings that did not provide sufficient analyzable information. Studies published outside the predefined time interval were excluded, with the exception of selected historical references included solely for contextual purposes.

The exclusion of non-English studies was adopted for feasibility and consistency of full-text assessment and synthesis and is acknowledged as a limitation of the review.

### 2.4. Study Selection

All records identified through the database searches were exported to Zotero [9], and duplicate records were removed prior to screening. The remaining records were screened in two sequential stages. First, titles and abstracts were assessed for thematic relevance. Second, the full texts of potentially eligible articles were evaluated against the predefined inclusion and exclusion criteria.

During title and abstract screening, records were excluded if they were clearly unrelated to the review question, did not address Galectin-3, or were not relevant to the predefined disease categories. At the full-text stage, articles were excluded based on the pre-defined criteria.

**Figure 1:**
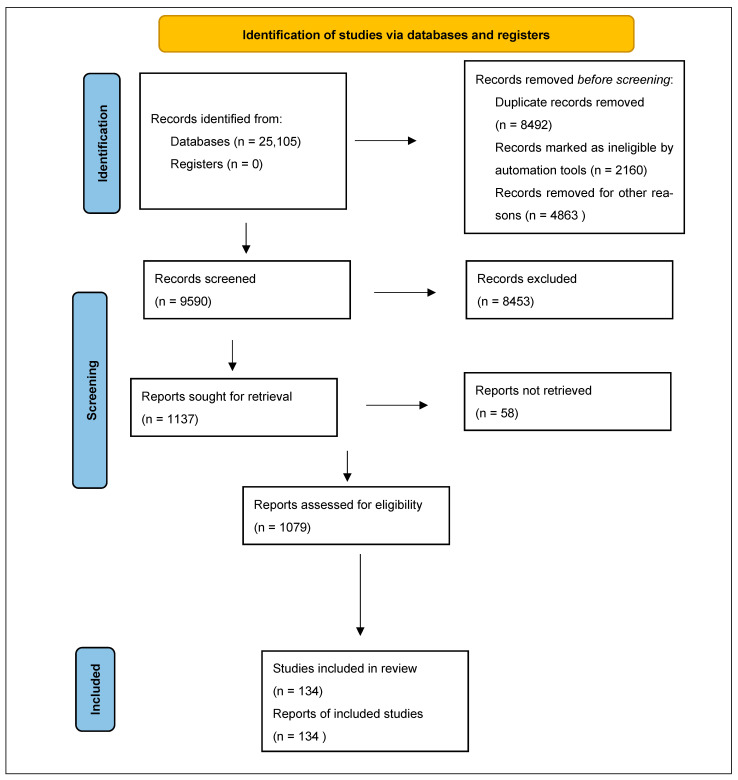
PRISMA flow diagram illustrating the study identification, screening, eligibility assessment, and inclusion process.

The study selection process is summarized in the PRISMA flow diagram (Figure 1). A total of 25,105 records were identified through database searching across three electronic databases (PubMed = 2436; MDPI = 769; Google Scholar = 21,900). Prior to screening, 8492 duplicate records were removed using Zotero, 2160 records were marked as ineligible by automation tools, and 4863 records were removed based on the pre-defined exclusion criteria, leaving 9590 records for title and abstract screening. Of these, 8453 were excluded at the screening stage, and 1137 reports were sought for retrieval. A total of 58 reports could not be retrieved, leaving 1079 reports assessed for full-text eligibility. Of these, 945 were excluded for different reasons including lack of focus on Galectin-3, publication outside the predefined time interval, unavailability of full text, non-English language, and inappropriate article type. Finally, 134 studies were included in the qualitative synthesis.

### 2.5. Information Sources and Search Strategy

The systematic literature search was conducted using three electronic databases: PubMed, Google Scholar, and the MDPI platform. These sources were used in a complementary manner to retrieve literature relevant to Galectin-3 across the predefined disease categories addressed in this review. To avoid duplicate counting, records available through publisher-hosted platforms were not considered independent entries when the same studies had already been identified through the broader search process.

The search was restricted to studies published between January 2020 and June 2025, in order to focus on recent evidence regarding the role of Galectin-3 in human disease. A limited number of earlier landmark publications were retained exclusively to provide historical and biological context in the Introduction; these contextual references were not included in the PRISMA-based eligibility assessment or study selection process.

The search strategy employed Boolean operators (AND, OR) to combine free-text terms, adapted to the interface of each database.

PubMed: [Title/Abstract] field tags were applied to ensure search precision. The complete set of search strings applied in PubMed, together with the number of records retrieved for each query, is presented in Table 1. It should be noted that these search strings were executed independently and their results are not mutually exclusive; a considerable proportion of the disease-specific records are subsets of the 2436 records retrieved by the general search, and overlaps across disease-specific searches are likewise expected. Accordingly, the aggregate of individual search results does not represent the total number of unique records retrieved from PubMed.

MDPI: the same disease-specific search strategy was applied using simplified Boolean operators (AND, OR), without field tags, adapted to the MDPI search interface (as presented in Table 2).

Google Scholar: The same disease-specific search strategy was applied using Boolean operators (AND, OR), with quotation marks used around (as presented in Table 3) multi-word phrases to ensure search precision, adapted to the Google Scholar interface. As with the previous searches, these strings were executed independently and their results are not mutually exclusive. All searches were restricted to publications from January 2020 to June 2025.

It is important to interpret the results retrieved across the three databases with caution, as a substantial degree of overlap is expected between PubMed, Google Scholar, and the MDPI platform. Google Scholar, in particular, indexes a broad range of sources, including the majority of records available through both PubMed and MDPI, meaning that a considerable proportion of the retrieved records are likely to appear across multiple databases simultaneously. To address this issue, all records identified through the three databases were subsequently imported into Zotero (version 7.0, Corporation for Digital Scholarship), a reference management software that was employed to systematically detect and eliminate duplicate entries prior to the screening phase. The number of records remaining after deduplication was then taken forward for title and abstract screening in accordance with the predefined eligibility criteria. This step ensured that each unique record was assessed only once, regardless of the number of databases in which it had been identified, thereby preserving the integrity of the selection process.

### 2.6. Data Extraction

For each eligible study, the following data were extracted: first author, year of publication, disease category, study design, study population or sample type, comparator group when applicable, Galectin-3-related outcome, and the main diagnostic, prognostic, mechanistic, or therapeutic findings.

### 2.7. Data Synthesis

Because of the broad thematic scope of the review and the heterogeneity of the included literature, a quantitative meta-analysis was not performed. Instead, the findings were synthesized narratively and organized by disease category. The evidence was grouped into major clinical domains, including cardiovascular diseases, cancer, metabolic disorders, renal diseases, digestive diseases, endometriosis, neuroinflammation and neurodegenerative diseases, and ophthalmological conditions.

Particular attention was given to the diagnostic and prognostic value of Galectin-3, its mechanistic involvement in inflammation and fibrosis, and its emerging relevance as a therapeutic target.

## 3. An Overview of Galectins in Humans

Galectins comprise a family of endogenous glycan-binding proteins [10,11]. Glycan-binding proteins (GBPs) are constituents of plants (seeds/lectins), animals/humans (immune cells/tissue), and pathogens (viruses/bacteria) [12].

These proteins were first discovered in the mid-1970s. Subsequently, in 1994, they were named “Galectins” with the corresponding numbers attached in the order that they were discovered [13]. Their structure is very diverse. Galectins belong to the lectin family, which also includes other types of lectins called C-type lectins, P-type lectins, and L-type lectins, depending on their structure or binding properties [14]. Galectins contribute to many cellular functions such as immune surveillance and apoptosis [10]. They are involved in cell–cell interactions, cell surface signaling, cell adhesion, intracellular processes, and cell–extracellular matrix (ECM) interactions. The known extracellular ligands of Galectin-3 are fibronectin, integrin, laminin, Mac-2 binding protein (colon adenocarcinoma) [15], and glycoproteins containing β-galactoside in the ECM and cell surface.

Galectins are found intracellularly or extracellularly in almost all organisms and have many roles. These proteins regulate basic cellular functions such as growth, proliferation, inflammation, cell–cell or cell–matrix interactions, and differentiation. Galectin-3 is one of the most studied members of the galectin family, and it has multiple physiological and non-physiological roles depending on the biological context. Many studies have been published on the role of Galectin-3 in cardiovascular diseases, kidney disorders, digestive tract and accessory gland disorders, gynecological conditions, and immunity diseases (Table 4). Galectin-1 is another frequently studied member alongside Galectin-3. In cancer, elevated expression of galectins is associated with tumor progression, such as high expression of Galectin-1, -3, or -8 in the nervous system (brain cancer), Galectin-3, -7, or -8 in the endocrine system (thyroid), and Galectin-1 or -4 in the respiratory system (lung cancer) and digestive system (esophageal and gastric cancer) [16]. In other disease contexts, low galectin expression is correlated with neoplastic tissue generation, such as the low expression of Galectin-7 in colon and pancreatic cancers and of Galectin-4 in liver cancer. Galectins can have potential protective roles such as Galectin-7 in gastric cancer and Galectin-9 in breast cancer. Galectin-3, -8, and -9 play a significant role in endothelial cell biology by modulating cell adhesion. They contribute to the penetration of circulating cells into tissues, tumor metastasis, and neo-tumor angiogenesis [17].

Studies have been conducted on the roles of Galectin-10 in immunity and respiratory disorders [18,19], and the roles of Galectin-13, Galectin-14, and the more recently discovered Galectin-16 in pregnancy [20,21,22,23].

Regarding Galectin-4, -8, -9, and 12, studies have investigated their involvement in various human disorders, such as respiratory, digestive, renal, metabolic, and immune disorders [20,21].

The galectin family is composed of three groups, which are classified according to their type of organization and the number of carbohydrate-recognition domains (CRDs). The prototype group includes those with a single CRD that can form homodimers, the chimera-type group includes those with a CRD and a “tail” to which it can bind, and the tandem-repeat group consists of four galectins, each with a CRD on one of the two protein domains [24].

**Figure 2:**
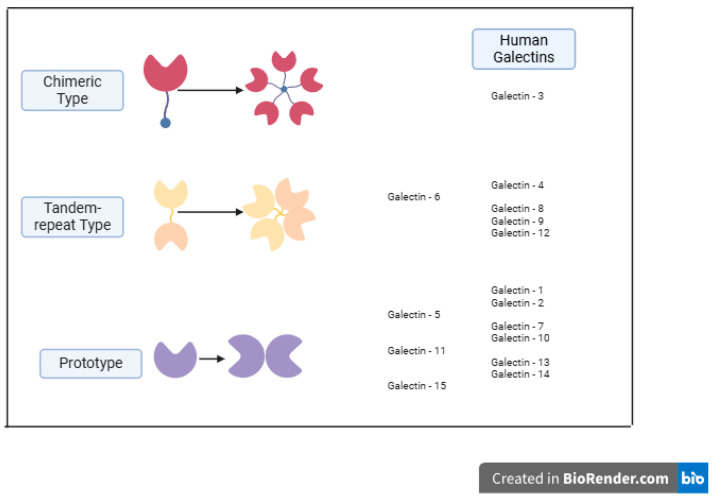
Galectin groups according to their structure, adapted from Mimura, S. et al., 2025 [26].

The most recently identified galectin is Galectin-16, and some studies have focused on its tissue specificity; however, its structure and the function that it plays remain poorly characterized [23]. In previous studies, LGALS16 was identified in placental tissue [25]. LGALS16, together with LGALS13 and LGALS14, is located on chromosome 19 in a cluster of human protein-coding genes [25] (Figure 2).

**Table 4:** Human galectins and the main groups of diseases with which they are associated. X—Mentioned in publications.

Galectin-1	**X**		**X**		**X**	**X**	**X**		**X**		**X**	**X**	**X**	**X**	**X**			[10,16,17]
Galectin-2								**X**		**X**								[14]
Galectin-3	**X**	**X**	**X**	**X**	**X**	**X**	**X**		**X**		**X**	**X**	**X**	**X**	**X**	**X**	**X**	[13,14,26,27,28,29,30,31]
Galectin-4					**X**			**X**	**X**	**X**			**X**		**X**			[14]
Galectin-7						**X**	**X**				**X**		**X**	**X**				[14]
Galectin-8								**X**		**X**	**X**		**X**		**X**			[14]
Galectin-9			**X**									**X**					**X**	[18,19,22]
Galectin-10					**X**												**X**	[16,17]
Galectin-12				**X**								**X**						[23,24]
Galectin-13																**X**		[20]
Galectin-14									**X**							**X**		[21]
Galectin-16																**X**		[21]
	Heart Failure	Myocardial Infarction	Renal Disease	MD	Respiratory system	Esophageal	Gastric	Recto-colon	Liver	Pancreas	Thyroid	Skin	Breast	Ovary	Prostate	Placenta	Immunity/Infections	
	Cardiovascular Disorders		Kidney Disorders	Metabolic Diseases	Respiratory Disorders	Digestive tract and accessory gland disorders					Endocrine Diseases	Skin Disorders	Reproductive System Disorders				Immune system	

Galectins are released extracellularly, where they will interact with glycoproteins at the cell surface or extracellular matrix [27] and be involved in responses to microenvironmental changes such as pH levels, hypoxia, and inflammation [26]. Galectins have various roles in humans, including involvement in physiological and pathogenic expressions, and exert various effects and functions depending on the biological environment. 

## 4. Characteristics of Galectin-3

Galectin-3 is a protein belonging to the lectin family, which binds to the β -galactoside structure, playing an important role in numerous biological processes in various organs via its carbohydrate-recognition domain [27,28]. Galectin-3 is also known as IgE-binding protein, Mac2, CP35, and CBP30. It is located on chromosome 14 in the locus q21-22 and is encoded by the LGALS3 gene [28]. The cellular location of Galectin-3 includes the cytoplasm, nucleus, cell surface, and extracellular environment. It is a key protein that plays an important physiological role in the regulation of cellular functions such as adhesion, differentiation, proliferation and apoptosis, as well as in inflammation and immunity [29].

Galectin-3 is widely distributed in many tissues in humans, including hematopoietic tissues, lymph nodes, the thymus, and the spleen [30]. Regarding its production, it is synthesized by immune cells such as mast cells, eosinophils, and macrophages, and it can be secreted extracellularly [30,31,32].

Galectin-3 plays an important role in cytokine releases of inflammatory cells, including activation and chemotaxis [33].

It has become an important potential biomarker to be included in future research [29]. Galectin-3 is implicated in the development of many pathological conditions, including cardiovascular diseases, heart failure, cancer, fibrosis [34,35], viral infection, and autoimmune diseases. Several authors have demonstrated that elevated levels of circulating Galectin-3 are correlated with chronic inflammatory diseases [36,37].

### 4.1. Structural Domains (CRD, N-Terminal)

Galectin-3 has been identified in murine peritoneal macrophages. It consists of 250 amino acid residues and has a relative molecular mass of about 29–35 kDa [38].

In humans, Galectin-3 demonstrates the following characteristics:

(a) A sole member of a chimeric group, being present in different cells such as chondrocytes, in lining epithelium on diverse structures, in cells and tissues [30,39];
(b) Being present in various immune cells in adults [40];
(c) Having physiological functions in different cellular processes such as adhesion, activation, growth, and differentiation [41];
(d) Playing an important role in cell interactions.

Galectin-3 is the only member in the chimera-type group and contains three domains: an N-terminal, a 100-amino-acid collagen-like sequence, and a spherical C-terminal CRD.

**Figure 3:**
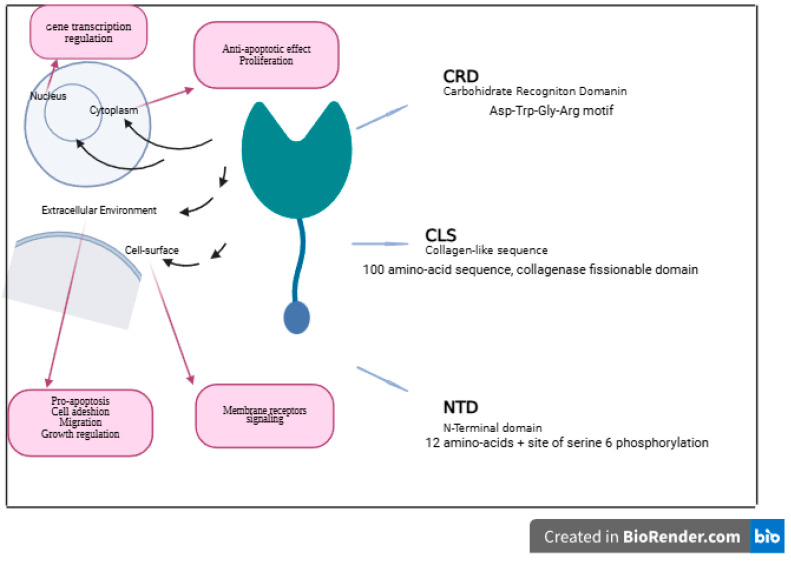
Structure of Galectin-3 with its intra- and extracellular domains, roles, and locations.

Galectin-3 binds to the cell surface through oligomerization via the carbohydrate-recognition domain [CRD] or N-terminal binding) [42] (Figure 3).

### 4.2. Roles, Activities, and Actions of Galectin-3

The physiological cell functions and pathological contexts of Galectin-3 are presented in Table 5:

(a) Regarding physiological cell functions, the expression of Galectin-3 is correlated with cell growth, tissue repair, cell differentiation, and cell adhesion [29,43,44].
(b) Regarding pathogenesis, Galectin-3 is involved in the development of fibrosis (cardiac fibrosis and atherosclerosis, which is the underlying cause of many cardiovascular diseases) [29]. It may also initiate and amplify inflammatory responses [29,45].

**Table 5:** Physiological cell functions and pathological context of Galectin-3.

**Physiological Cellular Functions of Galectin-3**	**References**
Cell growth	[29]
Cell proliferation	[29,46]
Cell apoptosis	[29,47]
Cell differentiation	[29]
Cell adhesion	[29,48]
Tissue repair	[29]
**Pathological implications of Galectin-3**	**References**
Atherosclerosis	[29,36]
Inflammation. ImmunityMetabolism	[17,23,31,49]
Organic fibrosis	[25]
Cancer	[50]

Galectin-3 also exerts contrasting functions, acting as both a proinflammatory mediator and an anti-inflammatory modulator. The expression of Galectin-3 in the extracellular matrix, on the cell surface, and in the cytoplasm, mitochondria, and nucleus underlies its many contrasting functions [37]. Galectin-3 can exert multiple and contradictory functions during inflammation depending on the cell type and context:

(a) As a mediator by stimulating proinflammatory cytokine production;
(b) As a modulator by suppressing immune cell activation [50].

Numerous studies have shown that this lectin acts as an inflammatory factor with a role in macrophage activation and intravascular inflammation [51].

## 5. Implications of Galectin-3 in Various Diseases

**Figure 4:**
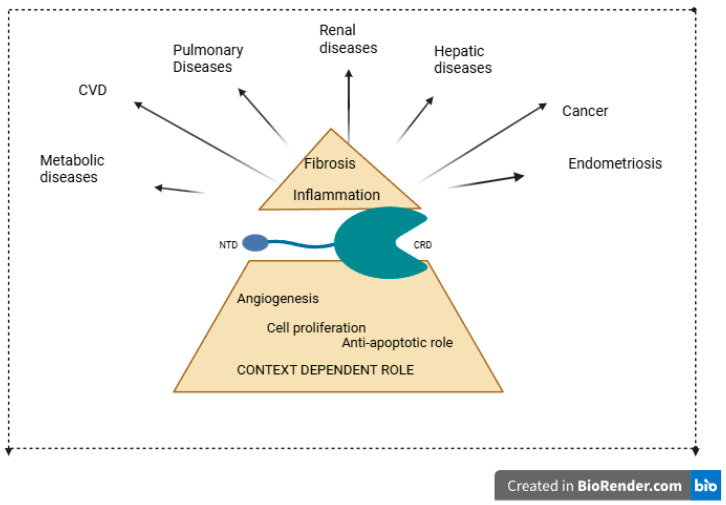
Schematic illustration of Galectin-3, showing its structural organization and its involvement in key physiological and pathological processes. Galectin-3 consists of an N-terminal regulatory domain, which mediates oligomerization and protein–protein interactions, and a C-terminal carbohydrate-recognition domain (CRD) responsible for β-galactoside binding. Through these domains, Galectin-3 regulates inflammation, cell proliferation, apoptosis resistance, angiogenesis, and fibrosis, and contributes to the pathogenesis of cardiovascular, metabolic, pulmonary, renal, hepatic, and neoplastic diseases.

Galectin-3 participates in various physiological and pathological processes such as inflammation, fibrosis, cell apoptosis, cell adhesion, angiogenesis, cell migration, cell proliferation, and cell differentiation [29]. In pathogenesis, it acts as an important player molecule (Figure 4).

Oxidative stress happens when there is an imbalance between the production and accumulation of oxygen reactive species (ROS) in cells and tissues and the organism’s ability to detoxify. Antioxidant enzymes play an important role in cell growth and health through cell metabolism. They degrade and eliminate reactive oxygen species, reactive nitrogen species (RNS), and free radicals. They are present in the mitochondria, cytosols, and other structures involved in the defense system [52,53,54]. The antioxidant enzymes superoxide dismutase, glutathione, and catalase act as the first line of defense, and damage to these enzymes is associated with the progression of chronic diseases, cancer, and neurodegenerative diseases [55,56]. Oxidative stress is considered an important contributor to diseases, and antioxidant defense is important in its prevention [57,58]. A high concentration of oxygen reactive species can mediate damage to the cell structure. Oxidative stress has been found to contribute to atherosclerosis, endothelial dysfunction, and cardiovascular diseases [59,60].

Based on recent studies, Galectin-3 (Gal-3) is not primarily acting as an antioxidant factor, but rather as a key mediator that increases oxidative stress and inflammation in pathological conditions. It is frequently described as a pro-oxidant that promotes reactive oxygen species (ROS) production, especially in cardiovascular disease and liver fibrosis [61,62,63]. Thus, Galectin-3 could generally function as a pro-oxidant/pro-inflammatory factor that accelerates tissue damage, and its inhibition is a therapeutic strategy to improve antioxidant status in chronic diseases [64,65,66].

### 5.1. Galectin-3 in Cardiovascular Diseases

Cardiovascular diseases are one of the leading causes of morbidity and mortality worldwide, although in many developed countries, the incidence rates have shown a declining trend [67], which is linked to advances in preventive measures and treatment. One of the main causes of cardiovascular diseases is atherosclerosis [45].

The prevention and treatment of cardiovascular diseases generate high medical and social costs. An innovative approach to the management of the cascade of cellular, molecular, and systemic events implicated in the development of cardiovascular diseases involves complex cardioprotective activities targeting several groups of biological, biochemical, inflammatory, and oxidative stress parameters. The aim is to interfere early with the biological mechanism underlying the initiation, generation, and complication of atherosclerotic plaque and to therapeutically manipulate this mechanism to achieve the most effective primary and secondary prevention of cardiovascular diseases.

Galectin-3, known as an essential mediator in the pathophysiology of cardiovascular diseases [28], promotes inflammation and cardiac fibrosis and is a biomarker with elevated levels in heart failure [45,68].

Atherosclerosis is a systemic endothelial dysfunction that occurs and develops in the foci of blood vessels and is accompanied by chronic inflammation and fibro-proliferation. It is a prothrombotic, angiogenic, and multifactorial disease that develops at the arterial level due to altered retention of low-density lipoproteins, hemodynamic disturbances, and oxidative stress. Atherosclerotic lesions tend to occur in predictable anatomical sites in the arterial tree (bifurcations, side branches, and areas with turbulent blood flow). In the eccentric region of an atheroma, particularly in relation to ischemia, blood vessel walls form, which can induce angiogenesis [69,70,71]. The concomitant chronic inflammation amplifies angiogenesis within the plaque, while endothelial cell dysfunction contributes to the prothrombotic state of the atherosclerotic plaque [69].

As an example, atrial fibrillation is a cardiac arrhythmia whose etiology includes oxidative stress and inflammatory reactions, which are evident in its progression.

#### 5.1.1. Atherosclerosis

Atherosclerosis is the cause of many cardiovascular diseases, including ischemic heart disease, coronary artery disease, and cerebrovascular disease [29]. Galectin-3 has a predictive importance for myocardial dysfunction developed after acute myocardial infarction [72,73,74]. A previous study involving 89 patients with preserved left ventricular ejection demonstrated that in the post-MI period, there were no statistically significant differences in Galectin-3 levels across the three examined groups on days 1, 5, and 30. The authors found that the initial levels of Galectin-3 correlated with atherosclerosis factors. On day 30, Galectin-3 levels correlated with diastolic dysfunction [75].

#### 5.1.2. Heart Failure (HF)

Heart failure results from impaired heart pump function, and its incidence is increasing worldwide [45,76]. Diagnosing heart failure based on clinical presentation alone is difficult, as the symptoms may be nonspecific. Paraclinical evaluation is therefore necessary in these cases. When a patient suspected of HF is clinically evaluated, medical history and chest X-ray results are important, but angiography or echocardiography is also necessary to assess left ventricular ejection function (LVEF). Worldwide, HF societies defined HF as a cardiac syndrome with functional and structural cardiac alterations. These clinical evaluations are supplemented by laboratory test results and biomarkers that can help determine the type of respiratory or cardiac dyspnea. Biomarkers already established for cardiovascular assessment, such as B-type natriuretic peptide, N-terminal pro-BNP, and troponin, are vital factors when assessing patients, but other biomarkers are also needed for a more comprehensive assessment [29,45,76,77]. High levels of natriuretic peptide (NP) are identified in HF patients. Galectin-3 is a cardiovascular biomarker approved by the Food and Drug Administration [78]. It can be a very useful biomarker for patients with left ventricular ejection fraction (LVEF) or FEVG [68,75,79,80]. Studies also confirmed that Galectin-3 is a prognostic predictive biomarker in acute onset HF patients [81].

#### 5.1.3. Atrial Fibrillation

Atrial fibrillation is the most common cardiac arrhythmia among heart diseases. It elevates the risk for other diseases such as heart failure, myocardial infarction, and chronic kidney disease, and it is also associated with diabetes, hypertension, and aging [82]. Specific interactions and implications of Galectin-3 in atrial fibrillation remain underexplored [83]. In this context, Galectin-3 has emerged as a relevant studied lectin because of its established involvement in extracellular matrix remodeling, fibroblast activation, and inflammatory signaling. Recent evidence suggests that elevated Galectin-3 levels are associated not only with the presence and persistence of atrial fibrillation, but also with atrial remodeling and recurrence after rhythm-control interventions, supporting its potential diagnostic, prognostic, and predictive value in this setting [84,85,86].

### 5.2. Galectin-3 in Cancer

Abnormal expression of galectins has been identified in different types of cancers, including in their development, progression, and metastasis [87,88,89]. Oncology is currently at the forefront of medicine, and the early detection and treatment of cancer is central to biomedical research. Tumor development is known to involve genetic mutations. The link between cancer and abnormal glycosylation was first reported 50 years ago, and recent studies have also demonstrated the role of epigenetic alterations in addition to genetic mutations [90], as well as the association of Galectin-3 expression with mitochondrial metabolism and glycolysis, with Galectin-3 mediating tumor cells’ metabolic adaptation to local hypoxia and reduced nutrient availability. According to Kim et al., malignant cell transformations related to Galectin-3 include tumor growth, cell adhesion and motility, angiogenesis, and pro-apoptosis or anti-apoptosis. In malignant tumors, Galectin-3 levels are elevated, and this elevation is correlated with the progression of the neoplasm or the possibility of metastasis. A translocation of Galectin-3 from the nucleus to the cytoplasm during the evolution of prostate neoplasms could explain why reduced Galectin-3 expression is associated with altered nucleus/cytoplasm expression patterns [7]. In a study involving 223 patients with bladder cancer, Galectin-3 expression was demonstrated to be higher in T1-stage tumors and decreased in T2-stage tumors, highlighting its potential role in early tumor progression [91,92]. Pancreatic ductal adenocarcinoma is a pancreatic cancer with a poor prognosis and an overall survival rate of 5 years in less than 5–10% of patients [93]. A review published in 2023 by Dimitrijevic Stojanovic et al. explored the role of Galectin-3 in pancreatic ductal carcinoma, indicating that it has diverse roles in tumor microenvironment cell interactions and immune response modulation. The serum levels of Galectin-3 can have diagnostic or prognostic value in both malignant and benign pancreatic conditions [93]. Plasma levels of Galectin-3 are significantly higher in patients with multiple myeloma. In vitro expression has been correlated with increased resistance and invasion in multiple myeloma [94].

### 5.3. Galectin-3 in METABOLIC Diseases

Alongside cardiovascular diseases, metabolic disorders have emerged as a global health problem with the incidence rates estimated to be on the rise. The effects of Galectin-3 depend on the pathological context or its specific expression in cells and tissues. Galectin-3 is believed to be involved in the progression of cardiovascular and metabolic disorders through its inflammatory effects [95].

Galectin-3 regulates diverse biological processes and is involved in the pathogenesis of diseases related to chronic inflammation, such as diabetes and diabetic retinopathy. The role of Galectin-3 in renal diseases is still not completely understood [96]. Galectin-3 is upregulated across several metabolic conditions, including obesity, type 2 diabetes, and metabolic dysfunction-associated steatotic liver disease, where it contributes to chronic low-grade inflammation, insulin resistance, lipid dysregulation, and progressive tissue remodeling [97]. Diabetic retinopathy is the main cause of preventable blindness in adults worldwide. The retinal pigment epithelium plays a critical role in the pathogenesis of diabetic retinopathy [98,99]. In a human multi-omics association study, circulating Galectin-3 was linked to fasting insulin, the triglyceride-glucose index, lipoprotein-related metabolites, and inflammatory pathways, supporting its integration into broader networks of metabolic dysfunction rather than its role as an isolated marker [100]. In addition, Galectin-3 has been implicated in metabolic liver disease, particularly in the progression of steatosis-associated fibrosis, reinforcing the concept that this lectin functions not only as a biomarker but also as a mechanistic mediator of metabolic injury and fibrotic remodeling [101]. Taken together, these findings suggest that Galectin-3 represents a relevant bridge between inflammation, fibrosis, and metabolic disease progression.

### 5.4. Galectin-3 in Gastric Diseases

Pectin, a soluble dietary fiber, is crucial for colonic digestion processes. It is possible that Galectin-3’s CRD interacts with intestinal cells, but further research is needed on these possible connections and interactions [102]. Galectin-3 plays an important role in inflammatory bowel disease (IBD) by being involved in immune regulation and tissue remodeling [103,104]. Its expression is associated with NLRP3 inflammasome activation and is thus linked to chronic intestinal inflammation. It is also involved in the development of fibrosis, autoimmune hepatitis, and biliary cholangitis [50].

### 5.5. Galectin-3 in Liver Diseases

The serum expression of Galectin-3 is higher in patients with hepatocellular diseases, such as toxic hepatitis and alcoholic or non-alcoholic cirrhosis, than in healthy people [105,106]. Galectin-3 is associated with tumor angiogenesis and metastasis in the progression of hepatocellular carcinoma [91]. Galectin-3 has low expression in hepatocytes under physiological conditions, but it is overexpressed in hepatocellular carcinoma and cirrhosis [107]. It plays a role in promoting the proliferation and activation of hepatic stellate cells [26]. Galectin-3 could be a promising therapeutic target in liver diseases considering its roles in inflammatory processes in acute intravascular hemolysis, which can lead to liver injury due to the accumulation of hemolysis products [108,109,110,111].

Cirrhotic cardiomyopathy is a disease characterized by abnormal cardiac function in the context of cirrhosis [112,113]. Its multifactorial pathogenesis comprises the abnormal function and structure of various substances including lectins. The inhibition of Galectin-3 can be a potential treatment target for this disease [114].

Galectin-3 plays an important role in fibrogenic and inflammatory mechanisms associated with chronic liver and metabolic diseases. Modulation of inflammatory pathways related to Galectin-3 represents a potential therapeutic strategy for metabolic and hepatic disorders. Bioactive dietary compounds, such as polyphenols, may influence Galectin-3-mediated inflammatory pathways involved in gastric, metabolic, and liver diseases [115].

### 5.6. Galectin in Respiratory Diseases

The literature suggests that galectins play a crucial role in respiratory diseases. As reported in a previous study, Gal-10 is involved in asthma through the formation of Charcot–Leyden crystals [116]. Beyond this mechanism, galectins participate more broadly in airway inflammation, immune-cell activation, tissue remodeling, and fibrotic responses across a range of pulmonary disorders. Recent evidence indicates that Galectin-3 is implicated in the pathophysiology of asthma, chronic obstructive pulmonary disease, acute lung injury, and other pulmonary conditions, where it contributes to inflammatory signaling and structural remodeling of the respiratory tract [117,118].

By mediating viral attachment that facilitates viral entry and modulating the immune response, Galectin-3 also plays a crucial role in local inflammation and tissue damage. In a study on SARS-CoV-2 and influenza A virus, Galectin-3 is mentioned as being involved in viral adhesion and penetration and the subsequent modulation of the immune response [119]. In addition, clinical evidence from patients with COVID-19 acute respiratory failure suggests that increased Galectin-3 levels are associated with more severe respiratory outcomes, including higher mortality risk, ICU admission, and more severe acute respiratory distress syndrome, further supporting its relevance in virus-associated pulmonary injury [120].

### 5.7. Galectin-3 in Kidney Diseases

The role of Galectin-3 in renal disorders has been evaluated in both preclinical and clinical studies. Studies have demonstrated an association between Galectin-3 and kidney diseases and have evaluated it as a potential biomarker for patient monitoring and therapeutic strategies [121,122]; elevated levels of Galectin-3 may be a prognostic factor or indicator of kidney damage, including acute kidney injury, but further studies are still needed to confirm the potential benefits of inhibiting its expression. Both the role of Galectin-3 as a biomarker in kidney diseases and its potential as an important therapeutic factor for the development of drug inhibitors have been studied. Some studies also evaluated the role of Galectin-3 in acute kidney injury. Two different evaluation studies showed that the level of Galectin-3 gradually increased with the severity of decline in renal function [42]. As mentioned above, aside from liver injuries, the prevention of inflammation in acute intravascular hemolysis could potentially prevent subsequent fibrosis and the development of liver fibrosis and chronic kidney diseases [108].

### 5.8. Galectin-3 in Endometriosis

Endometriosis is a benign, chronic inflammatory disease with an incidence of approximately 10% in women of childbearing age; it is characterized by the presence of stromal cells and endometrial glands outside the uterus [123,124]. These pelvic inflammatory changes may be asymptomatic or symptomatic, resulting in severe pelvic pain and impaired female fertility. The activity of Galectin-3 is influenced by the cellular environment’s pH, and even a small modification in the pH level can affect its activity and anti-inflammatory or proinflammatory properties [125,126]. As indicated by Hisrich et al., galectins can be potential biomarkers or therapeutic targets for future studies on gynecological diseases such as endometriosis.

### 5.9. Galectin-3 in Neuroinflammation and Neurodegenerative Diseases

In neuropathology, Galectin-3 has important cell surface and extracellular functions. In their review, Srejovic et al. concluded that Galectin-3–TLR4 interaction is necessary to induce inflammation. In animal model studies, the deletion of Galectin-3 attenuates the prion disease condition in mice, while in Alzheimer’s disease prone mice, Galectin-3 deletion improves cognitive functions [127].

Galectin-3 is involved in cognitive processes. Studies published on this topic have indicated the possible correlations of Galectin-3 with neuroinflammation and neurodegeneration. The mechanisms linking cognitive impairments related to aging or neurodegenerative diseases with Galectin-3 are unclear. Cognitive dysfunctionality is present in patients with hepatic encephalopathy, and Galectin-3 inhibitors have been confirmed as demonstrating therapeutic potential in non-alcoholic steatohepatitis, liver fibrosis, and cirrhosis. The serum levels of Galectin-3 in patients with type 2 diabetes mellitus and mild cognitive impairment have been shown to be higher compared with controls [102].

Recent studies have demonstrated that Galectin-3 contributes to neurodevelopment, microglial activation, and remyelination. In a study by Tan et al., Galectin-3 measured using peripheral blood tests was found to be a potential risk factor for Alzheimer’s disease. This finding warrants further clinical studies and investigations for therapeutic purposes, as it is associated with the onset of this disease [128].

Galectin-3 has also been studied in optic neuritis, a neuropathology associated with multiple sclerosis. Some animal model studies have shown that Galectin-3 is associated with experimental autoimmune encephalomyelitis pathogenesis, as its expression is identified in the microglia optical pathway, indicating the role of Galectin-3 in the pathogenesis of optic neuritis and its potential as a therapeutic target [129].

### 5.10. Galectin-3 in Ophthalmological Conditions

In ophthalmology, it is important to conduct research in areas related to the prevention and treatment of degenerative eye diseases, dry eye syndrome and inflammation, and retinal disorders such as macular degeneration and diabetic retinopathy. Numerous studies have reported that Galectin-3 is associated with retinal detachment and light-related retinal degeneration [96,130,131,132,133,134].

Factors that contribute to oxidative stress, which leads to the development and progression of chronic heart, kidney, and liver diseases, as well as metabolic disorders including type 2 diabetes, can also play significant roles in ocular conditions such as diabetic neuropathy, cataracts, dry eye syndrome, age-related macular degeneration, and neurodegeneration [132,135,136,137,138,139].

According to Ito et al., Galectin-3 levels in the tears of patients with vernal keratoconjunctivitis may be a marker of corneal epithelial damage [140]. A case–control study by Hata-Mizuno et al. in 2022, with 28 patients and 14 controls, found a correlation between the concentration of Galectin-3 in tears and the severity of dry eye syndrome [141].

## 6. Future Research Directions

Inhibition of Galectin-3 in inflammatory and fibrotic diseases has been shown to have a therapeutic effect. Further studies are necessary to determine the exact role of Galectin-3 in tissue injuries in acute intravascular hemolysis.

Inhibition of Galectin-3 can also be a promising strategy in clinical investigations on Alzheimer’s disease prevention. Further research into the structure and connections of this lectin is also extremely useful for the development of therapies for many diseases, particularly cancers.

The study of the biology and biochemistry of this protein could lead to the development of a possible candidate for future therapy targets, as it is involved in tumor progression and metastasis, apoptosis, and various inflammatory processes. This study provides new insights into the roles and functions of Galectin-3. As shown in Table 4, there is widespread interest in human galectins due to their roles in pathophysiology.

Galectin-3 has attracted considerable interest as a potential biomarker in various diseases, particularly cardiovascular diseases or cancer. The evidence for this role has correlational and prognostic values, not causal. There is also interest in its potential as a prognostic, staging, or diagnostic factor. The study of Galectin-3 has sparked significant interest in the fields of pharmaceuticals. A research direction included new perspectives in precision medicine to understand the comorbidity mechanism of cancer and atherosclerosis.

There are few testing studies using preclinical or clinical trials regarding small- and large-molecule Galectin-3 inhibitors. The drug biostability and polar surfaces of the small molecules allow us to design smaller structures (Table 6).

Galectins may be important targets for new drug discovery due to implications in inflammation, cancer or several diseases. Various studies are underway that consider glycomimetic molecules through different approaches like potential drugs for modulating or binding galectins [148].

## 7. Conclusions

Galectin-3 has a pleiotropic protein role. It is involved in many physiologic and pathophysiological pathways. This narrative review presents an overview of the roles of galectins, particularly Galectin-3, and their pathophysiological implications in various clinical conditions, indicating their role in specific conditions.

Galectin-3, as a member of the lectin family, has a pleiotropic physiological role and is involved in numerous pathogenetic pathways. It plays an important role in fibrosis development and different inflammation-based diseases.

Many studies included Galectin-3 as a prominent risk biomarker in plasma controls for prognosis in cardiovascular diseases and in heart failure.

Blood levels can also act as a risk marker for Alzheimer’s disease, which has important effects on neuroinflammation. Certain studies indicate the importance of Galectin-3 for therapeutic strategy purposes, highlighting that further clinical investigations are needed.

Although the serum level of Galectin-3 is associated with an increased risk of certain liver diseases, such as cirrhosis and liver failure, more research is needed to determine its potential as a therapeutic target or biomarker.

Further research is needed on the mechanism underlying the involvement of Galectin-3 in human health and disorders or associated complications. Therefore, in the medical field, Galectin-3 is emerging as a promising target for drug development.

## Figures and Tables

**Table 1:** PubMed search strings and records retrieved (January 2020–June 2025).

Disease Category	PubMed Search String	n
General search	(“Galectin-3”[Title/Abstract])	2436
Cardiovascular diseases	(“Galectin-3”[Title/Abstract]) AND (“cardiovascular diseases”[Title/Abstract])	51
Renal/kidney diseases	(“Galectin-3”[Title/Abstract]) AND (“renal diseases”[Title/Abstract] OR “kidney diseases”[Title/Abstract])	15
Metabolic disorders	(“Galectin-3”[Title/Abstract]) AND (“metabolic disorders”[Title/Abstract] OR “metabolic syndrome”[Title/Abstract])	34
Cancer/tumor/malignancy	(“Galectin-3”[Title/Abstract]) AND (“cancer”[Title/Abstract] OR “tumor”[Title/Abstract] OR “malignancy”[Title/Abstract])	579
Endometriosis	(“Galectin-3”[Title/Abstract]) AND (“endometriosis”[Title/Abstract])	7
Hepatic/liver diseases	(“Galectin-3”[Title/Abstract]) AND (“hepatic diseases”[Title/Abstract] OR “liver diseases”[Title/Abstract])	21
Respiratory/pulmonary diseases	(“Galectin-3”[Title/Abstract]) AND (“respiratory diseases”[Title/Abstract] OR “pulmonary”[Title/Abstract])	127
Neuroinflammation/neurodegenerative diseases	(“Galectin-3”[Title/Abstract]) AND (“neuroinflammation”[Title/Abstract] OR “neurodegenerative diseases”[Title/Abstract])	108
Ophthalmological/eye diseases	(“Galectin-3”[Title/Abstract]) AND (“ophthalmological”[Title/Abstract] OR “eye diseases”[Title/Abstract])	3

**Table 2:** MDPI search strings and records retrieved.

Disease Category	MDPI Search String	n
General search	Galectin-3	769
Cardiovascular diseases	Galectin-3 AND cardiovascular diseases	61
Renal/kidney diseases	Galectin-3 AND renal diseases OR kidney diseases	21
Metabolic disorders	Galectin-3 AND metabolic disorders OR metabolic syndrome	11
Cancer/tumor/malignancy	Galectin-3 AND cancer OR tumor OR malignancy	769
Endometriosis	Galectin-3 AND endometriosis	7
Hepatic/liver diseases	Galectin-3 AND hepatic diseases OR liver diseases	9
Respiratory/pulmonary diseases	Galectin-3 AND respiratory diseases OR pulmonary	18
Neuroinflammation/neurodegenerative diseases	Galectin-3 AND neuroinflammation OR neurodegenerative diseases	301

**Table 3:** Google Scholar search strings and records retrieved.

Disease Category	Google Scholar Search String	n
General search	“Galectin-3”	21,900
Cardiovascular diseases	“Galectin-3” AND “cardiovascular diseases”	7160
Renal/kidney diseases	“Galectin-3” AND (“renal diseases” OR “kidney diseases”)	2030
Metabolic disorders	“Galectin-3” AND (“metabolic disorders” OR “metabolic syndrome”)	5440
Cancer/tumor/malignancy	“Galectin-3” AND (“cancer” OR “tumor” OR “malignancy”)	15,600
Endometriosis	“Galectin-3” AND “endometriosis”	590
Hepatic/liver diseases	“Galectin-3” AND (“hepatic diseases” OR “liver diseases”)	2050
Respiratory/pulmonary diseases	“Galectin-3” AND (“respiratory diseases” OR “pulmonary”)	1600
Neuroinflammation/neurodegenerative diseases	“Galectin-3” AND (“neuroinflammation” OR “neurodegenerative diseases”)	6100
Ophthalmological/eye diseases	“Galectin-3” AND (“ophthalmological” OR “eye diseases”)	396

**Table 6:** Galectin-3 main inhibitors or modulators and their principal outcomes and considerations in studies and in preclinical or clinical phase trials. MCP—Modified Citrus Pectin (MCP), Gal-3—Galectin-3 and MCP-N—a Modified Citrus Pectin fraction [15,142,143,144,145,146,147,148].

Target Disease Context	Main Inhibitors or Modulators Developed	Main Results, and Safety or Considerations	References
Liver fibrosis	GB1107 synthetic inhibitor	Studies Signaling of liver damage—experimental approach to induced liver fibrosis in mice; GB 1107 reduced liver fibrosis—may be a therapeutic target	[142]
Angioneurotic sclerosis Cancer	Common pathways regulated by Gal-3	Studies Atherosclerosis: migration and proliferation, proinflammatory factor and endothelial damage Cancer: proliferation, invasion, metastasis	[143]
Bladder tumor	MCP Gal-3 antagonist	Studies Inhibited bladder tumor growth through downregulation of Galectin-3.	[144]
Antitumor activity in vivo and in vitro	GCS-100 (MCP)	Binding and antagonizing Galectin-2	[15,145]
Inhibit Galectin-3 in Cancer progression	Ph—MCP	MCP-N and degraded products—potential inhibitors for Galectin-3	[15,146]
Potential use applications for the development of functional foods or medicines	Rhamnogalacturonan-I containing pectic polyzaccharide	Isolated from pumpkin Potential developed for Galectin-3 inhibitor. Moderate binding affinity.	[15,147]

## Data Availability

Data is contained within the article.
